# Perioperative Factors Contributing the Post-Craniotomy Pain: A Synthesis of Concepts

**DOI:** 10.3389/fmed.2017.00023

**Published:** 2017-03-01

**Authors:** Tumul Chowdhury, Rakesh Garg, Veena Sheshadri, Lakshmi Venkatraghavan, Sergio Daniel Bergese, Ronald B. Cappellani, Bernhard Schaller

**Affiliations:** ^1^Department of Anesthesiology and Perioperative Medicine, University of Manitoba, Winnipeg, MB, Canada; ^2^Department of Anesthesiology, Pain and Palliative Care, Dr. BRAIRCH, All India Institute of Medical Sciences, New Delhi, India; ^3^Department of Anesthesiology, Toronto Western Hospital, Toronto, ON, Canada; ^4^Department of Anesthesiology, The Ohio State University, Wexner Medical Center, Columbus, OH, USA; ^5^Department of Research, University of Southampton, Southampton, UK

**Keywords:** pain, craniotomy, neurosurgical, intracranial, factors

## Abstract

The perioperative management of post-craniotomy pain is controversial. Although the concept of pain control in non-neurosurgical fields has grown substantially, the understanding of neurosurgical pain and its causative factors in such a population is inconclusive. In fact, the organ that is the center of pain and its related mechanisms receives little attention to alleviate distress during neurosurgical procedures. In contrast to the old belief that pain following intracranial surgery is minimal, recent data suggest the exact opposite. Despite the evolution of various multimodal analgesic techniques for optimal pain control, the concern of post-craniotomy pain remains. This paradox could be due to the lack of thorough understanding of different perioperative factors that can influence the incidence and intensity of pain in post-craniotomy population. Therefore, this review aims to give an in-depth insight into the various aspects of pain and its related factors in adult neurosurgical patients.

## Introduction

In the last 20 years, perioperative pain management has gained utmost importance, not at least because patient satisfactions parallel has risen to an absolute priority in health care systems. However, perioperative pain management in neurosurgical patients (especially the intracranial procedures) continues to be a gray area due to the existing controversies and lack of consensus regarding the standardized treatment. Many therapeutic recommendations are still based on anecdotal case reports and show a questionable success. However, in contrary to the earlier belief that the pain following intracranial surgery is minimal, some newer data suggest the exact opposite, so that newer and more scientifically based treatment modalities have to be taken into account (1).

Importantly, on the one hand, neurosurgical patients form a unique subgroup in which systemic manifestation of poorly controlled pain may negatively impact on the hemostasis and cerebral hemodynamics, therefore, leading to devastating conditions (2–4). On the other hand, overzealous use of analgesics can also affect the rapid neurological assessment and may result in increased morbidity (2–4). Besides these facts, the literature related to postoperative pain management in neurosurgical patients is mainly limited to the pharmacological aspects; however, the role of other conditions including the size of the incision, underlying pathology, surgical factors/methods used, experience of the surgeon, assessment tools used, and preoperative and intraoperative factors should also be emphasized to give a more balanced view of the topic.

In the last years, there could be found several controversies in the perioperative pain management of neurosurgical patients. This narrative review primarily aims to elucidate and summarize these various perioperative factors related to pain incidence and intensity in adult craniotomy procedures to give a basis for better and especially more differentiated clinical decisions in perioperative pain management.

## Incidence

On the contrary to general belief, patients underwent a craniotomy experienced a substantial amount of pain both in occurrence and intensity (2). Post-craniotomy pain incidences are reported from 30 to 90%, depending upon various perioperative factors (4–9). This significant variation is multifactorial and also represents a lack of well-organized prospective epidemiological studies in this domain (6). The majority of post-craniotomy pain incidences were reported with 48 h of surgery (6). Though the severity of pain declines as the time passes, there can still be a peak of moderate to severe pain on the second postoperative period (10, 11). In the same report, the pain in first 24 h postoperatively was observed in 93% of patients, and the severe pain was absent after the eighth postoperative day. Indeed, the moderate to severe pain can be found in approximately half of the patients after an hour of procedure (2). The incidence subsequently decreased 3% for each year of life (12). The impact of pain after a craniotomy has been variously reported due to non-uniformity in defining craniotomy pain and the surgical approaches. Strikingly, the site-specific craniotomy pain (acoustic neuroma surgery) is also described and presents in a significant number of postoperative patients (4, 5, 13). Rimaaja et al. reported 64% incidence of such pain and about one-third of patients revealed both pre- and postoperative headache (13). Post-craniotomy pain after supratentorial craniotomy ranges from 17.5 to 29.3% (14–16). Also, the reported incidence of a chronic headache after neurosurgery reduces with time after surgical intervention (14–17). This variation in incidence shows that, besides other perioperative complications, the headache has a unique feature, which has various origins.

## Mechanisms and Types of Pain

The variability in incidence and diagnosis of post-craniotomy pain can be better understood by taking in account the anatomical structures involved. The neural supply of scalp is from branches from cervical plexus and trigeminal nerve (3, 18). The supraorbital and supratrochlear nerves and divisions of the frontal nerve (ophthalmic division of trigeminal nerve) innervate the anterior portion of the scalp (3, 18). The zygomaticotemporal (maxillary division of trigeminal nerve), temporomandibular, and auriculotemporal nerves (mandibular division of trigeminal nerve) supply the temporal scalp (3). The cervical plexus branches, including greater auricular, and the greater, lesser, and least occipital nerves innervate the occipital scalp. The branches accompanying meningeal arteries innervate the dura mater. The free nerve endings and nociceptors are responsible for pain (3). Mechanical nociceptors that are connected to myelinated A-delta afferent nerve fibers (fast conducting, low threshold for activation) are activated with a stimulus-like sharp, pricking in nature. The unmyelinated, slow C-fibers are activated by mechanical, chemical, and cold–hot stimuli *via* polymodal nociceptors (3). There are several inflammatory/nociceptive mediators that regulate the pain type, origin, and intensity. It is still a complex and poorly understood phenomenon that some patients in comparison to others exhibit profound pain. Whether or not the amount or types of mediators play important role in this context is yet to be explored. The role of pharmacogenomics in post-craniotomy pain is another area of future research.

The craniotomy pain is primarily somatic and originates from the scalp, pericranial muscles, and soft tissue. The manipulation of the dura mater during surgical intervention also activated pain pathway (6). The physical stimulation caused by incision and traction utilized in craniotomy stimulates nervous terminals and specific nociceptors resulting in postoperative pain. The post-craniotomy pain is usually localized to the incision site and surrounding soft tissues. The generalized diffuse headache usually originates from dura (9, 14). The nature of pain after craniotomy is pulsating or usually pounding (19). The constant and continuous nature of pain is infrequently seen.

The persistent post-surgical pain after craniotomy is observed in many patients, and the proposed hypothesis includes dural irritation, pericranial muscle retraction, surgical trauma, decreased cerebrospinal fluid pressure, and aseptic meningitis (4). The patient position during the surgical intervention may lead to persistent tension headache and neck muscle spasm with possible muscular origin (19). For example, the mechanism of chronic persistent pain after acoustic neuroma resection is related to adherence of the dura to overlying muscles and has been related to surgical closure techniques (4, 20). Given such etiology for persistent pain, modified surgical closure such as replacement of the bone flap during the retrosigmoid approach to resection of vestibular schwannomas has been advocated. Such a modification has been found to reduce the rate of chronic headaches from 94 to 27% (5). Other modifications include fat grafting during closure with reduction of pain incidence from 30 to 12% (21). The role of cranioplasty with methyl methacrylate instead of craniectomy is controversial with regard to resolution of persistent pain (22, 23). Interestingly, in one study, more than 45% of post-craniotomy patients showed a headache during dental evaluation (17). These patients revealed tenderness around masseter muscles, and opening the mouth and jaw protrusion were the inciting events for a headache (17).

The literature shows that there is not one nature of postoperative pain, but there are several, sometimes small, conditions that contribute to such an event so that the variations of expressions of the pain can be explained.

## Perioperative Factors [Demographic/Surgical/Pathological/Anesthetic]

Factors such as the surgical site and technique, nature of the surgical intervention, gender, tumor type and size, muscle resection, and use of steroids have been studied for their association with incidence and severity of pain (2, 4). The literature reports are conflicting and inconclusive.

### Age and Gender

Whether or not the age and gender can influence post-craniotomy pain is a matter of conflicting evidence (2, 11, 24). A study suggests that there exists a gender difference in regard to early post-craniotomy pain after supratentorial brain tumor surgery. In this study, the female patients showed higher pain scores within 1 h of surgery (25). Similarly, the postoperative pain was found to be more common in females and younger patients; however, the role of psychological aspects was also taken into account (6). There exists a reverse relationship between the age and the postoperative pain (14). In another study, there was no association found between the gender difference and the post-craniotomy pain (2), which was supported by other studies as well (12, 26). The female gender is also linked to chronic pain (13, 14).

### Surgical Site

The influence of surgical site on craniotomy pain is also investigated. In this regard, the infratentorial procedures have been reported to have more severe pain as compared to supratentorial interventions (4, 27). Similarly, Gottschalk et al. found that patients with infratentorial procedures showed more pain scores in both rest and movement in first 2 days postoperatively (8). Strikingly, in a study comparing pain scores between intracranial and extra-cranial (lumbar spine surgery, mandible/maxillary fixation), the pain scores were less in intracranial surgeries (28). Thibault et al. studied six surgical sites and their effects on craniotomy pain and revealed that frontal site showed the least pain (29). Regression analysis in this study highlighted that the surgical site was the independent factor related to pain intensity (29).

### Surgical Intervention/Technique

Surgical intervention/technique has been shown to influence the pain associated with a craniotomy. In comparison to craniectomy, the pain after craniotomy is found to be less. In this study, other factors including demographic data and tumor size were comparable (24). Similarly, when cranioplasty after the craniotomy for posterior fossa surgery was compared with no cranioplasty-associated craniotomy, the former group showed fewer pain scores (30). Schaller and Baumann also investigated in posterior fossa surgery that a headache was substantially higher (94 versus 27%) in patients who did not have bone flap replaced (5). The severity of postoperative pain is also influenced by the extent of temporalis and posterior cervical muscles resection (31). Apparently, the larger resection would lead to more inflammation and more pain eventually. During the acoustic neuromas resection, the translabyrinthine approach is associated with less persistent pain as compared to retrosigmoid or suboccipital approach (4, 20–22, 32). The other preventive measures for a post-craniotomy headache during retrosigmoid approach are the use of adipose tissue, cranioplasty, and residue trapping (27, 28). Importantly, despite cranioplasty, the patients with suboccipital approach showed the persisted worse pain scores for a year (32). However, another study highlighted that postoperative pain would be minimal in retrosigmoid approach in the absence of intradural drilling (33). Also, surgical complications are also linked to worse post-craniotomy pain (11). This can be partly due to more tissue damage/handling.

### Psychological Factors

Preoperative anxiety and depression are the common psychological factors associated with post-craniotomy pain. These two factors are also linked to chronic pain as well (14). Relation of a headache can also be reciprocal with depression (13). The study found that the somnolence was also related to the intensity of postoperative pain (14). Interestingly, in the same study, patients may present a different type of headaches in pre- and postoperative periods. Preoperative pain can also influence the intensity and type of postoperative pain. Klimek et al. showed that the patients (in comparison to patients with no pre-existing pain) with pre-existing pain experienced sharper and referred pain postoperatively (11).

### Tumor Characteristics

Few studies also highlighted the relationship between tumor size and post-craniotomy pain. Strikingly, a study by Rimaaja et al. showed that the small size tumor was associated with chronic postoperative headache (13). On the contrary, Mosek et al. showed that the tumor size was not a predictor of postoperative pain (24, 34).

### Anesthetic Agents

Whether or not the type of anesthetics (TIVA versus volatile) can influence intensity and occurrence of post-craniotomy pain is questionable. A study by Mordhorst et al. showed that in comparison with TIVA, the sevoflurane-based anesthesia was associated with higher post-craniotomy pain intensity. Also, the pain was persisted for a longer period in the volatile anesthetic subgroup (12). This may be partly explained by the fact that the TIVA-based anesthetic produces less neuro-inflammation and hence less pain. An Italian multicenter trial investigated that propofol–remifentanil group incited less stress response and required fewer opioids consumption in supratentorial craniotomy (35). A recent study also supported this hypothesis and showed that the propofol-based anesthesia produced more anti-inflammatory mediators (IL-10) in neurosurgical procedures (36).

### Other Factors

Other relevant variables including the movement, obesity, smoking, alcoholism, use of steroid, presence of surgical drains, position of the patient, application of skull pins, rotation of the neck, comorbidities, and anesthetic/surgical duration and surgical experience may or may not influence the occurrence and intensity of post-craniotomy pain; however, there is no substantial evidence for these (11, 29). Among these, the use of preoperative steroid (due to its anti-inflammatory property) has a strong association with lesser post-craniotomy pain (2, 12). The other important factor is the role of pre-emptive analgesia on the post-craniotomy pain that has been given much attention in various papers; thereby, this section is not included in the present paper.

This summary of the different factors contributing to the postoperative pain is critical: according to of the law of requisite variety of Ashby, only “variety can destroy variety.” This theorem is particularly the case in pain management, as there have to be different treatment approaches to various pain origins.

## Effects of Pain on Cerebral Hemodynamics

Pain is known to have its impact on all body systems and organs. Cardiovascular effects include an increase in pulse rate and blood pressure. Hemodynamic fluctuations are undesirable during craniotomy procedures (37, 38). The precipitating factors for inducing sympathetic nervous activity have been correlated with plasma catecholamine concentrations as a surrogate marker (39). Hypertension during craniotomy is commonly associated with increased norepinephrine levels and activation of the renin–angiotensin–aldosterone system (39, 40). Fluctuations in blood pressure in patients with impaired cerebral autoregulation may impact cerebral blood flow and thus perioperative morbidity and mortality (4). Such hemodynamic fluctuations are frequently observed during scalp incision, periosteal detachment, dural opening, and brain retraction (38, 41, 42). This mandates in time hemodynamic stabilization for neurosurgical procedures and optimal pain management. Thus, appropriate management can help to reduce the rate of various adverse reactions and complications of surgery including hemorrhage, premature aneurysmal rupture, and cerebral ischemia caused by suboptimal pain control and hemodynamic fluctuations. The other associated problem with poor pain management is to use more opioids, which in turn increases the risk of postoperative nausea and vomiting. In addition, the inadequate control may also cause increased chances of postoperative cognitive dysfunction, agitation, and delirium. Importantly, these postoperative problems are usually seen as complications of neurosurgery and often; the pain management for such patients does not receive immediate attention.

## Conclusion

It is evident that the common perception of the post-craniotomy pain is changing, and indeed, it is a severe postoperative problem that is sometimes difficult to manage as well. However, in-depth understanding of various perioperative factors related to pain occurrence and intensity can impart better patient’s management. Future investigation should reveal such factors in more detail through the well-designed studies.

## Author Contributions

TC and RG have given substantial inputs for developing the concept, design, data collection, synthesis, interpretation, and writing the manuscript. VS and LV have given important inputs for developing the concept and design and writing the manuscript. SB, RC, and BS have assisted in writing and editing the manuscript.

## Conflict of Interest Statement

The authors declare that the research was conducted in the absence of any commercial or financial relationships that could be construed as a potential conflict of interest.
